# Characterization of a *Paracoccidioides* spp. strain from southeastern Brazil genotyped as *Paracoccidioides restrepiensis* (PS3) and review of this phylogenetic species

**DOI:** 10.1590/1678-4685-GMB-2019-0201

**Published:** 2020-05-29

**Authors:** Tiago Alexandre Cocio, Erika Nascimento, Marcia R. V. Z. Kress, Eduardo Bagagli, Roberto Martinez

**Affiliations:** 1 Universidade de São Paulo (USP), Faculdade de Medicina de Ribeirão Preto (FMRP), Departamento de Clínica Médica, Ribeirão Preto, SP, Brazil.; 2 Universidade de São Paulo (USP), Faculdade de Ciências Farmacêuticas de Ribeirão Preto (FCFRP), Departmento de Análises Clínicas, Toxicológicas e Bromatológicas, Ribeirão Preto, SP, Brazil.; 3 Universidade Estadual Paulista ‘Júlio Mesquita Filho’ (UNESP), Instituto de Biociências de Botucatu, Departamento de Ciências Químicas e Biológica, Botucatu, SP, Brazil.

**Keywords:** Paracoccidioides restrepiensis, *Paracoccidioides brasiliensis* PS3, phylogenetic species, evolution, paracoccidioidomycosis epidemiology

## Abstract

Phylogenetic species of *Paracoccidioides brasiliensis* complex (S1a and S1b, PS2, PS3, and PS4) and *Paracoccidioides lutzii* are agents of paracoccidioidomycosis, an endemic fungal disease in Latin America. *P. restrepiensis* (PS3 genotype) was classified as monophyletic and geographically restricted to Colombia and neighboring territories. BAT (or Pb-327B) was isolated from a patient living in the southeast region of Brazil but with genotype similar to Colombian *Paracoccidioides* spp. strains. This study aimed to define the phylogenetic species of BAT isolate by using additional genotyping methods, as well as reviewing the epidemiological and clinical studies related to *P. restrepiensis* isolates. Genomic DNA of BAT isolate and reference strains of *P. brasiliensis sensu stricto* (S1b), *P. americana* (PS2), *P. restrepiensis* (PS3), and *P. lutzii* were analyzed by conventional polymerase chain reaction (PCR) of partial *gp43* exon 2 loci, by PCR-RFLP technique of *tub*1 gene, and by sequencing of the whole *gp43* exon 2 loci. Here, we show that BAT isolate belongs to *P. restrepiensis* species, which is an unusual identification in southeastern Brazil, where *P. brasiliensis sensu stricto* is the prevalent genotype. This identification has relevance for geographical distribution and propagation of the genus *Paracoccidioides* in South America.

## Introduction

Paracoccidioidomycosis (PCM) is a systemic fungal infection endemic and restricted to Latin American countries such as Brazil, Argentina, Colombia, and Venezuela (Martinez, 2017). Pathogens that cause the acute and chronic forms of PCM are thermodimorphic fungi belonging to the genus *Paracoccidioides*, family *Ajellomycetaceae*, order *Onygenales*, class *Eurotiomycetes*, and species *Paracoccidioides brasiliensis* and *Paracoccidioides lutzii* (Gonzalez and Hernandez, 2016). *P. brasiliensis* clade is composed of five phylogenetic species, in which S1a and S1b belong to the paraphyletic group distributed in Brazil, Argentina, Paraguay, Peru, and Venezuela; PS2 belongs to the monophyletic group distributed in Brazil and Venezuela; PS3 belongs to the monophyletic group found mainly in Colombia; and the PS4 monophyletic group is found exclusively in Venezuela (Matute *et al.*, 2006; Carrero *et al.*, 2008; Teixeira *et al.*, 2009; Teixeira *et al.*, 2014; Muñoz *et al.*, 2016). Turissini *et al.* (2017) analyzed microsatellites, mitochondrial and nuclear genes, proposing four new species belonging to the genus *Paracoccidioides*: *P. brasiliensis sensu stricto* (S1a and S1b), *P. americana* (PS2), *P. restrepiensis* (PS3), and *P. venezuelensis* (PS4). These species show among them genotypic and micromorphological divergences (Turissini *et al.*, 2017). The *P. lutzii* clade contains exclusively *P. lutzii* (Teixeira *et al.*, 2009).

Phylogenetic species 3 (PS3), now *P. restrepiensis*, was characterized by Matute *et al.* (2006) and classified as monophyletic, geographically restricted to Colombia, and considered an evolutionary lineage independent of other phylogenetic species of *Paracoccidioides* spp. complex. The same authors described the phylogenetic relationship of *P. restrepiensis* (PS3) with other species of *P. brasiliensis* complex, showing ancestral proximity to *P. brasiliensis sensu stricto* (S1a and S1b), but having a greater genetic distance from *P. americana* (PS2). Munõz *et al.* (2016), when analyzing genotypic divergences among the phylogenetic species, verified the ancestral proximity of Colombian *P. restrepiensis* (PS3) isolates with Venezuelan isolates of *P. venezuelensis* (PS4) and Argentinian and Brazilian isolates of *P. brasiliensis sensu stricto* (S1a and S1b). Besides the genetic proximity of *P. restrepiensis* (PS3) to other phylogenetic species of *P. brasiliensis* complex, Roberto *et al.* (2016) characterized two strains (human isolate chronic form PCM, and soil isolate) obtained in the Venezuelan territory as PS3 (now *P. restrepiensis*), suggesting its regional dissemination in South America.

This study aimed to characterize a clinical isolate from southeastern Brazil as *P. restrepiensis* (PS3), an unusual finding in such geographical area. Additionally, a review has been presented with studies on human and environmental isolates of the same genotype.

## Material and Methods

### *Paracoccidioides* spp. isolates and culture conditions

BAT (also known as Pb-327-B) clinical strain was isolated in 1985 from a suppurated lymph node of a patient resident in a city belonging to the metropolitan region of Ribeirão Preto, São Paulo State, Brazil (21º10’13.44” S and 47º48’37.17” W). The patient was a 33-year-old male rural worker who had the subacute form of PCM manifested by generalized lymphadenomegaly, hepatosplenomegaly, disseminated cutaneous lesion, fungal lesions in duodenal and colonic mucosa, and jaundice. The patient denied previous disease history or travel to other Brazilian states and South American countries. PCM diagnosis was supported by *Paracoccidioides* spp. isolation in culture, histopathological examination of intestinal lesions, and a 1:1024 serum titer in the counterimmunoelectrophoresis for anti-*Paracoccidioides* spp. antibodies. The patient obtained clinical cure after two years of treatment with sulfa drugs.

The following reference strains, whose genotypes were determined in other studies, were employed for BAT clinical isolate comparison: Pb 18 – representative of *P. brasiliensis sensu stricto* (S1b) species (Matute *et al.*, 2006); Pb dog-EPM 194-representative of *P. americana* (PS2) species and T2-EPM 54-representative of *P. restrepiensis* (PS3) species (Roberto *et al.*, 2016); and Pb 01 representative of *P. lutzii* (Teixeira *et al.*, 2009). All the strains are maintained by successive subcultures on Sabouraud Agar Dextrose medium (Oxoid) plus 0.15 g l^-1^ chloramphenicol sodium succinate (Blau Farmacêutica), and incubated at 25 °C. The study was approved by the Research Ethics Committee of the Hospital das Clínicas of Ribeirão Preto Medical School, University of São Paulo (Protocol HCRP nº 4456/2017).

### Genomic DNA extraction of *Paracoccidioides* spp. strains

The genomic DNA of *Paracoccidioides* spp. strains were obtained from the fungal mycelia, which were grown in a synthetic modified McVeigh-Morton liquid medium for 35 days at 25 °C in an orbital shaker at 130 rpm (Infors HT-Ecotron) (Restrepo and Jimenez, 1980). The mycelia were subjected to extraction of genomic DNA according to the method I (treated glass beads and phenol-chloroform-isoamyl alcohol), with minimal modifications (van Burik *et al.*, 1998). The genomic DNA was treated with 300 ng ml^-1^ RNase A^®^ (Thermo Fisher Scientific) at 37 °C for one hour. The concentration of genomic DNA was determined by using NanoDrop 2000^®^ (Thermo Fisher Scientific) and its integrity checked in 1% agarose gel using SYBR^®^ Safe DNA gel stain (Thermo Fisher Scientific) and visualized using the ChemiDoc XRS+ imager with Image Lab software (Bio-Rad).

### Partial *gp43* exon 2 loci PCR amplification

To identify and classify BAT clinical isolate into the genus *Paracoccidioides*, the genomic DNA of *Paracoccidioides* spp. reference strains and BAT isolate were submitted to partial amplification of the *gp43* exon 2 loci by using the primers gp43-E2F: (5- CCA GGA GGC GTG CAG GTG TCC C – 3) and gp43-E2R: (5- GCC CCC TCC GTC TTC CAT GTC C – 3) (Cisalpino *et al.*, 1996; Roberto *et al.*, 2016) at 10 mM concentration, and annealing temperature at 58 °C. PCR reaction was performed with Taq polymerase enzyme-GoTaq® Green Master Mix (Promega) according to the manufacturer’s instructions. The final volume of PCR reaction was 25 μl, containing 500 ng genomic DNA. Thermocycling was performed in the Vapo Protect^®^ thermocycler (Eppendorf). PCR products, approximately 533 bp, had their integrity verified in 2% agarose gel by using SYBR® Safe DNA gel stain (Thermo Fisher Scientific). Its molecular weight was determined by 100-bp Ladder marker, Ready-To-Use (Sinapse), and visualized and photographed on the ChemiDoc XRS+ imager with Image Lab software (Bio-Rad).

### Polymerase Chain Reaction – Restriction Fragment Length Polymorphism of *tub*1 gene – PCR-RFLP

Phylogenetic species identification of BAT clinical isolate was made according to Roberto *et al.* (2016). Briefly, PCR-RFLP of alpha-tubulin (*tub*1) gene was performed with Taq polymerase-GoTaq® Green Master Mix enzyme (Promega). The final volume of PCR reaction was 25 μl, containing 500 ng genomic DNA and the primers *tub1*F: (5-CTG GGA GGT ATG ATA ACA CTG C-3) and *tub1*R: (5- CGT CGG GCT ATT CAG ATT TAA G -3) (Kasuga *et al.*, 2002; Roberto *et al.*, 2016) at a concentration of 10 mM, and annealing temperature of 58 °C. PCR *tub1* products (263 bp) were cleaved with *Bcl*I and *Msp*I endonucleases (Thermo Fisher Scientific) at a concentration of 10 U μL^-1^ each at 37 °C per 16 hours, according to manufacturer’s instructions. Cleaved DNA fragments were visualized in 2.5% agarose gel at 70 V for 140 minutes in presence of SYBR® Safe DNA gel stain (Thermo Fisher Scientific) and 50 bp DNA ladder molecular marker (Sinapse) and compared according to the method described by Roberto *et al.* (2016).

### Sequencing of *gp43* exon 2 loci

To validate the *tub1* gene PCR-RFLP method, *gp43* exon 2 loci was sequenced using the primers *Pbgp43-E2F:* (5-CTA GAA TAT CTC ACT CCC AG-3) and *Pb_gp43-E2R:* (*5-*GCC CCC TCC GTC TTC CAT GTC C*-3*) (Cisalpino *et al.*, 1996; Hrycyk *et al.*, 2018) at a concentration of 20 mM and annealing temperature of 58 °C. PCR product of *gp43* exon 2 loci, approximately 722 bp, had nonspecific amplification and/or integrity verified in 2% agarose gel. Then PCR amplicons were purified using Wizard^®^ SV Gel and PCR Clean-Up System kit (Promega), as instructed by the manufacturer. DNA sequences were determined with an ABI3730^®^ DNA Analyzer (Applied Biosystems), using the BigDye^®^ Terminator v3.1 Cycle Sequencing Kit (Thermo Fisher Scientific). Chromatograms were analyzed with ChromasPro^®^ software (ChromasPro 2.6.5). The DNA sequences were compared to nucleotide database using the Basic Local Alignment Search Tool (blastn): https://blast.ncbi.nlm.nih.gov/Blast.cgi (Altschul *et al.*, 1990). The sequences of *gp43* exon 2 loci determined in this study were submitted to alignment and analysis of similar and conserved regions, using Clustal Omega software https://www.ebi.ac.uk/Tools/msa/clustalo/ (Sievers *et al.*, 2011).

BAT clinical isolate sequence of *gp43* exon 2 loci was deposited at GenBank: (https://www.ncbi.nlm.nih.gov/genbank/) under accession number MH484614.

## Results

### BAT clinical isolate belongs to the genus *Paracoccidioides*

**Figure 1:**
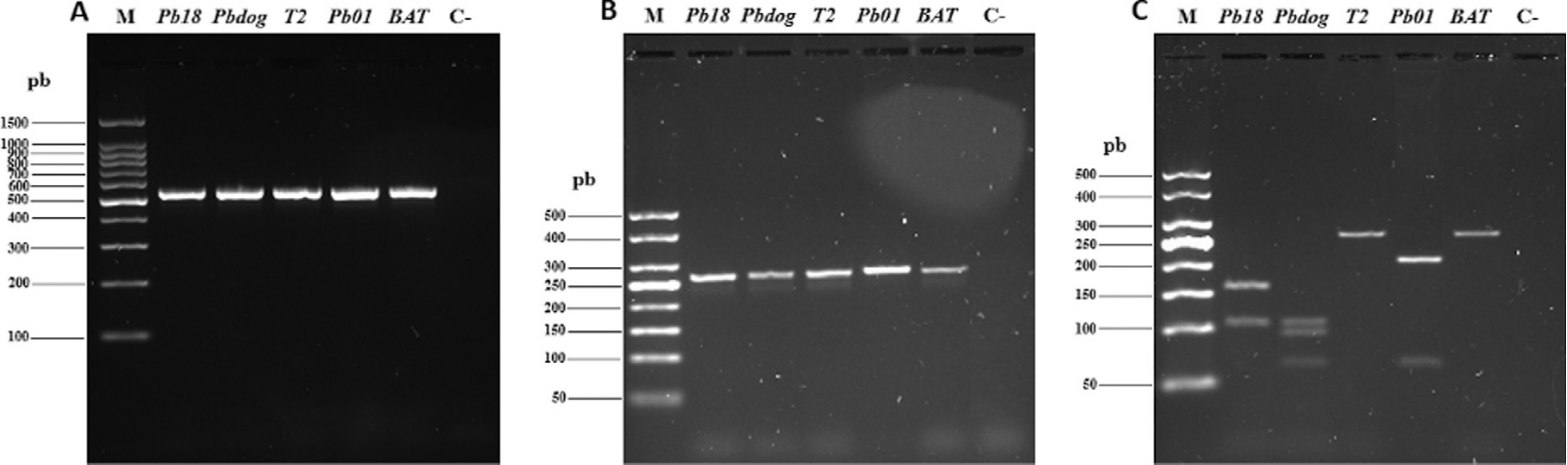
(A) Partial amplification of the *gp43* exon 2 loci of *Paracoccidioides* spp. by conventional PCR of BAT clinical isolate and reference isolates. M: 100 bp DNA ladder molecular marker, Ready-To-Use (Sinapse® Inc., United States). (B) Amplification of the *tub1* gene by PCR-RFLP. M: 50 bp DNA ladder molecular marker (Sinapse® Inc., United States). (C) PCR-RFLP DNA fragment patterns obtained after cleavage with *Bcl*I and *Msp*I endonucleases, showing similarity of BAT clinical isolate with T2-EPM54 (*P. restrepiensis* – PS3 reference strain). M: 50 bp DNA ladder molecular marker (Sinapse® Inc., United States). Pb 18: *P. brasiliensis sensu stricto* (S1b); Pb dog-EPM 194: *P. americana* (PS2); T2-EPM 54: *P. restrepiensis* (PS3); and Pb 01: *P. lutzii*; BAT: Clinical isolate under study.

Genomic DNA from *Paracoccidioides* spp. reference strains and BAT isolate were subjected to standard PCR to partially amplify *gp43* exon 2 loci. A PCR product of approximately 533 bp (Figure 1A) was observed, confirming that all study samples, including BAT clinical isolate, belong to the genus *Paracoccidioides*.

### Molecular characterization of BAT clinical isolate as *P. restrepiensis* (PS3)

**Figure 2:**
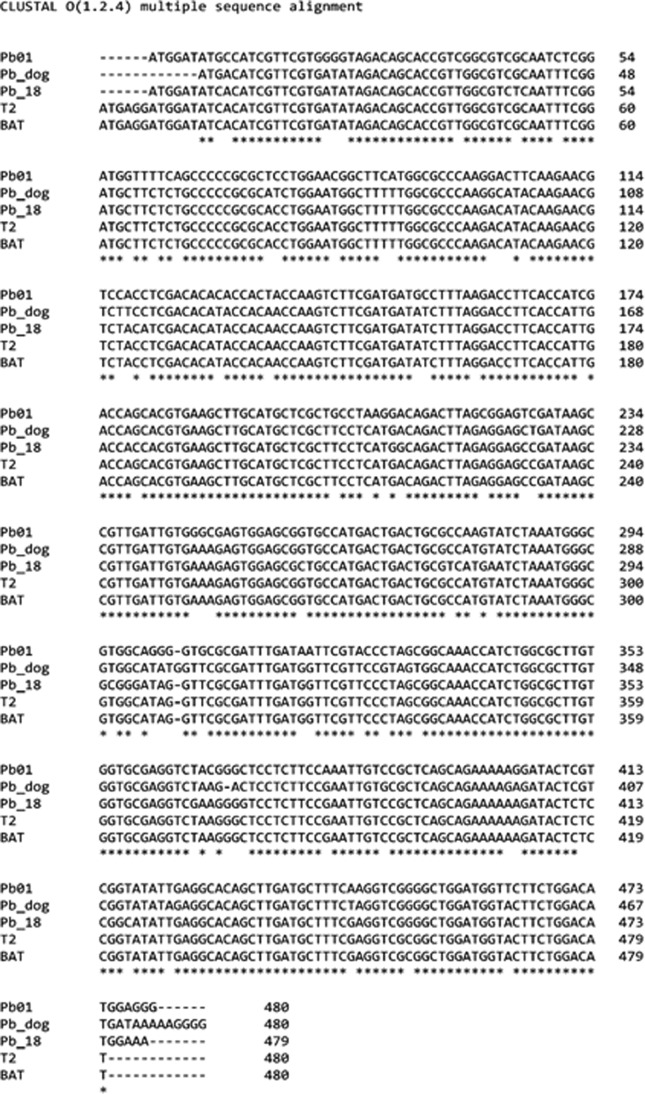
Alignment of *gp43* exon 2 loci nucleotide sequences from *Paracoccidioides* spp. reference strains and BAT clinical isolate. The identity between BAT isolate and T2-EPM54 (*P. restrepiensis* – PS3 reference strain) was observed, as well as genetic proximity with Pb 18 (*P. brasiliensis sensu stricto* – S1b) but with greater phylogenetic distance from Pb dog-EPM194 (*P. americana* – PS2) and Pb 01 (*P. lutzii*). *: represents similar nucleotides in the analyzed sequences.

The *tub*1 gene of *Paracoccidioides* spp. reference strains and BAT isolate were PCR amplified, and 263 bp amplification products were observed (Figure 1B). BAT clinical isolate was identified as *P. restrepiensis* (PS3) (Figure 1C) since the 263 bp fragment of the *tub*1 gene does not have cleavage sites for *Bcl*I and *Msp*I endonucleases; thus, it was maintained in its complete integrity (263 bp). The reference strains Pb 18, Pb dog–EPM194, T2 – EPM54, and Pb 01 had DNA fragment patterns produced by the endonucleases, as described by Roberto *et al.* (2016), validating the molecular identification of BAT isolate species by PCR-RFLP as *P. restrepiensis* (PS3) genotype (Figure 1C). The whole *gp43* exon 2 loci of BAT clinical isolate was sequenced to confirm the result obtained by PCR-RFLP. The *gp43* exon 2 loci DNA sequence showed 100% identity for nucleotide sequence of the reference strain T2-EPM54 *P. restrepiensis* (PS3). Alignment of nucleotide sequences of the *gp43* exon 2 loci of BAT clinical isolate and the reference strains also showed genetic proximity with Pb 18-*P. brasiliensis sensu stricto* (S1b), but greater phylogenetic distance from Pb dog EPM194 – *P. americana* (PS2) and from Pb01 (*P. lutzii*) (Figure 2).

**Figure 3:**
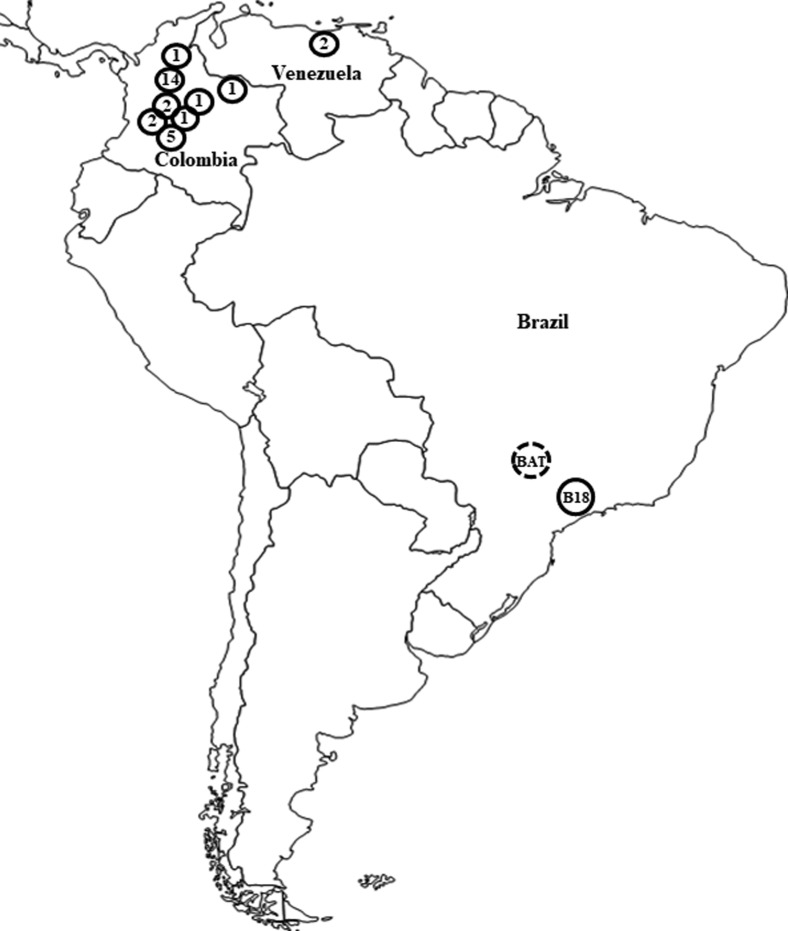
Geographic distribution of human and environmental isolates of *P. restrepiensis* (PS3 genotype) in South America. Each circle represents the identified isolates and their respective regions (Colombia and Venezuela) according to reports in the literature (Matute *et al.*, 2006; Muñoz *et al.*, 2016; Roberto *et al.*, 2016). In Brazil: BAT clinical isolate (PCM-subacute form) isolated from a patient in the macro-region of Ribeirão Preto, São Paulo State, Brazil, and B18 or Pb339 was isolated from a patient in the state of São Paulo, Brazil.

The geographical origin of BAT clinical isolate and of the other *Paracoccidioides* spp. isolates classified as *P. restrepiensis* (PS3 genotype) are shown in Figure 3.

## Discussion

BAT clinical strain was isolated in 1985 and maintained at the Ribeirão Preto Medical School-USP, southeastern Brazil. It was classified as *P. restrepiensis* (PS3) by sequencing of *gp43* exon 2 loci and PCR–RFLP of *tub*1 gene. This result was unexpected since phylogenetic species of PS3 (*P. restrepiensis*) have so far been isolated in Colombia and Venezuela, countries in northwestern South America. A sample of this strain was sent to Venezuela in the 1990s and included in two investigations on genetic diversity of *Paracoccidioides* spp. These studies showed a genotypic difference between BAT clinical isolate and other Brazilian isolates. First, BAT isolate (named Pb 327-B) was evaluated together with 32 *P. brasiliensis* clinical and environmental isolates from different South American countries. By employing randomly amplified polymorphic DNA (RAPD), BAT isolate was grouped near *P. brasiliensis* isolates from Colombia, which was later classified as PS3 genotype (Calcagno *et al.*, 1998). All 33 *P. brasiliensis* isolates but one had their DNA analyzed by restriction fragment length polymorphism (RFLP), using *Hin*fI and *Hin*cII endonucleases. The dendrogram showed a great relationship of BAT clinical isolate with Colombian *P. brasiliensis* strains (Nino-Vega *et al.*, 2000).

Some phenotypic characteristics of BAT isolate were evaluated. Micromorphological aspects, virulence for guinea pig, and serological reaction of BAT-isolate exoantigen with rabbit anti GP43 serum were typical traits of *P. brasiliensis* strains (Lacaz CS *et al.*, 1999). Compared to other *P. brasiliensis* isolates, BAT isolate had high exoantigen production, which was recognized by sera from patients with PCM in southeastern Brazil (Silva-Vergara *et al.*, 1998; Panunto-Castelo *et al.*, 2003). BAT clinical isolate has been used as a source of antigens of *Paracoccidioides* spp. in serological tests for PCM diagnosis, since it, together with antigens of other strains, formed a pool with high reactivity against sera from PCM patients from São Paulo state, Brazil (Vidal *et al.*, 2014). Sera from patients infected by *P. brasiliensis* S1 genotype reacted with BAT isolate exoantigen in immunodiffusion test (data not shown). Paracoccin, a 160 kDa Glc-NAc-binding lectin of the BAT isolate yeast wall, adhered to laminin and induced TNFα and NO production by macrophage cells (Coltri *et al.*, 2006).

To date, 31 *P. restrepiensis* (PS3) strains (clinical and environmental isolates) have been identified and reported in the literature. The studies encompassed phylogeny, molecular characterization, morphology, serology, and/or epidemiology (Table 1). The geographic distribution of isolates characterized as *P. restrepiensis* (PS3), including BAT clinical strain, is predominant in Colombia, totaling 87.2% of the 31 isolates, followed by 6.4% in Venezuela, and 6.4% in Brazil (Matute *et al.*, 2006; Muñoz *et al.*, 2016; Roberto *et al.*, 2016). The occurrence of *P. restrepiensis* (PS3) in other South American countries (Brazil and Venezuela) suggests that its geographical distribution is not restricted to the Colombian territory as initially presumed. It is believed that the phylogeographic characteristics of *P. restrepiensis* (PS3) are due to a possible and relatively recent biogeographic expansion of *P. brasiliensis sensu stricto* (S1a and S1b) to Colombia, associated to events of geographic barriers represented by the uplifting of the Andes mountain range and submersion of the Colombian territory by formation of the Pebas-Solimões lake (Wesselingh and Salo, 2006; Teixeira *et al.*, 2014). The emergence of *P. restrepiensis* (PS3) in Brazil was not clarified within the process of speciation of these phylogenies, due to absence of a geographical barrier in the territory (Teixeira *et al.*, 2014).

**Table 1:** *Paracoccidioides* spp. isolates genotypically characterized as *P. restrepiensis* (PS3): origin, source, molecular identification, and study type (1967 – 2018).

Strain		Origin	Source	Identification method	Study Type	Reference
Original ID	Other ID					
C1	P149	Colombia	Chronic /PCM	MLST	Phylogenetic	(Matute *et al*., 2006)
C2	P159	Antioquia, Colombia	Chronic /PCM	MLST	Phylogenetic	(Matute *et al*., 2006)
C3	P163	Antioquia, Colombia	Chronic /PCM	MLST	Phylogenetic	(Matute *et al*., 2006)
C4	ATCC 60855	Antioquia, Colombia	Chronic /PCM	MLST	Phenotypic	(Bustamante-Simon *et al*., 1985)
					Phenotypic	(Villar *et al*., 1988)
					Virulence	(Gomez *et al.*, 2001)
					Phylogenetic	(Matute *et al*., 2006)
C5	P141	Antioquia, Colombia	Chronic /PCM	MLST	Phylogenetic	(Matute *et al*., 2006)
C6	P196 / Higuita	Antioquia, Colombia	Chronic /PCM	MLST	Molecular Identification	(Corredor *et al*., 2005)
					Phylogenetic	(Matute *et al*., 2006)
C7	P204	Antioquia, Colombia	Chronic /PCM	MLST	Phylogenetic	(Matute *et al*., 2006)
C8	P292	Antioquia, Colombia	Chronic /PCM	MLST	Phylogenetic	(Matute *et al*., 2006)
C9	P68	Antioquia, Colombia	Chronic /PCM	MLST	Phylogenetic	(Matute *et al*., 2006)
C10	P72	Cordoba, Colombia	Chronic /PCM	MLST	Phylogenetic	(Matute *et al*., 2006)
C11	P46	Antioquia, Colombia	Chronic /PCM	MLST	Phylogenetic	(Matute *et al*., 2006)
C12	P161	Antioquia, Colombia	Chronic /PCM	MLST	Phylogenetic	(Matute *et al*., 2006)
**C13**	76533	Antioquia, Colombia	Chronic /PCM	MLST	Susceptibility	(Hoyos *et al*., 1984)
					Phylogenetic	(Matute *et al*., 2006)
C14	H31	Boyaca, Colombia	Chronic /PCM	MLST	Phylogenetic	(Matute *et al*., 2006)
C15	H45	Cundinamarca, Colombia	Chronic /PCM	MLST	Phylogenetic	(Matute *et al*., 2006)
C16	H47	Arauca, Colombia	Chronic /PCM	MLST	Phylogenetic	(Matute *et al*., 2006)
C17	P206	Antioquia, Colombia	Chronic /PCM	MLST	Phylogenetic	(Matute *et al*., 2006)
C18	P151	Antioquia, Colombia	Chronic /PCM	MLST	Phylogenetic	(Matute *et al*., 2006)
C19	CIB44197	Caldas, Colombia	Armadillo	MLST	Molecular Identification	(Corredor *et al*., 2005)
					Phylogenetic	(Matute *et al*., 2006)
C20	Pb73/ ATCC 32071	Antioquia, Colombia	Unknown	MLST	Serological tests	(Restrepo-Moreno 1967)
					Phylogenetic	(Matute *et al*., 2006)
C21	CIB40392	Caldas, Colombia	Armadillo	MLST	Epidemiological	(Corredor *et al*., 1999)
					Phylogenetic	(Matute *et al*., 2006)
T2	EPM54	Caracas, Venezuela	Soil	MLST, PCR – RFLP	Phylogenetic	(Roberto *et al*., 2016)
5598	EPM62	Caracas, Venezuela	Acute/PCM	MLST, PCR – RFLP	Phylogenetic	(Roberto *et al*., 2016)
JDA – 80	EPM77	Medellin, Colombia	Unknown	MLST, PCR – RFLP	Phylogenetic	(Roberto *et al*., 2016)
MSCol	EPM81	Medellin, Colombia	Chronic /PCM	MLST, PCR – RFLP	Phylogenetic	(Roberto *et al*., 2016)
I9	EPM83	Bogotá, Colombia	Chronic /PCM	MLST, PCR – RFLP	Phylogenetic	(Theodoro *et al*., 2008)
					Serological	(Machado *et al*., 2013)
					Proteome	(Pigosso *et al*., 2013)
					Secretome	(de Oliveira *et al*., 2018)
*B18	Pb339 or	Brazil	Unknown	MLST, PCR - RFLP	Serological tests	(Restrepo-Moreno 1967)
	ATCC 32069				Virulence	(Gomez *et al.*
	EPM01 or B339				Serological	(Camargo *et al*., 2003)
	or ATCC 200273				Phylogenetic	(Matute *et al*., 2006)
					Mitochondrial DNA	(Salgado – Salazar *et al*., 2010)
					Phylogenetic	(Roberto *et al*., 2016)
					Phylogenetic	(Muñoz *et al*., 2016)
PbBac	-	Colombia	PCM	Genomic sequencing	Phylogenetic	(Muñoz *et al*., 2016)
PbCnh	-	Colombia	Chronic /PCM	Genomic sequencing	Phylogenetic	(Muñoz *et al*., 2016)
PbJam	-	Colombia	Chronic /PCM	Genomic sequencing	Phylogenetic	(Muñoz *et al*., 2016)
BAT	Pb327 - B	Ribeirão Preto – São Paulo, Brazil	Subacute/PCM	MLST, PCR – RFLP	Serological	(Silva – Vergara *et al.*, 1998)
					Mycological	(Lacaz Cda, S *et al.*, 1999)
					Phylogenetic	(Calcagno *et al.*, 1998)
					Phylogenetic	(Niño – Vega *et al.*, 2000)
					Serological	(Panuto – Castelo *et al.*, 2003)
					Immunologic	(Coltri *et al.*, 2006)
					Molecular Identification	This Study

Besides the geographical origin of *P. restrepiensis* (PS3), the respective clinical manifestation of PCM is one of the important aspects for understanding the pathogenicity of this phylogenetic species. Among the 31 *P. restrepiensis* (PS3) isolates described in the literature, including BAT isolate, 25 (76%) strains were isolated from patients. The chronic form of PCM was more prevalent in patients infected with *P. restrepiensis* (PS3), representing 88% of the cases. Acute/subacute forms of PCM caused by *P. restrepiensis* (PS3) were only reported in two patients, including that with a disseminated disease from which the strain evaluated in this study was isolated (Matute *et al.*, 2006; Muñoz *et al.*, 2016; Roberto *et al.*, 2016). In a comparative study of PCM cases caused by *P. brasiliensis sensu stricto* (S1a and S1b) and *P. americana* (PS2) in Rio de Janeiro state-Brazil, the prevalence of chronic form was observed for both species (de Macedo *et al.*, 2019). The same was observed in a clinical and epidemiological study of PCM caused by *P. lutzii* in the state of Mato Grosso-Brazil, wherein all patients were diagnosed with the chronic form (Hahn *et al.*, 2019). In general, chronic form predominance is common in PCM, so it does not distinguish *P. restrepiensis* (PS3) genotype in this regard.

Some of the *Paracoccidioides* spp. isolates characterized as *P. restrepiensis* (PS3) have already had their biology studied (Matute *et al.*, 2006; Theodoro *et al.*, 2008). Genotypic and phenotypic studies for *P. restrepiensis* (PS3) isolates were fungal antigenicity (Restrepo-Moreno and Schneidau, 1967), ketoconazole susceptibility (Hoyos *et al.*, 1984), murine immune response to PCM, conidia morphology and sporulation at different culture media (Bustamante-Simon *et al.*, 1985), dimorphism (Villar *et al.*, 1988), morphological analysis and molecular identification of armadillo isolates (Corredor *et al.*, 1999), and melanin production (Gomez *et al.*, 2001). Other studies compared *P. restrepiensis* (PS3) isolates with different species belonging to *Paracoccidioides* spp. complex (*P. brasiliensis sensu stricto* (S1), *P. americana* (PS2)) to evaluate genotypic and phenotypic differences. Corredor *et al.* (2005) studied polymorphic genes (*gp43* exon 2 loci, ITS_1, and ITS_4) from clinical and armadillo strains isolated in the Colombian territory, comparing them with strains isolated in other South American countries. Polymorphic differences were found among genes when compared to strains identified as *P. brasiliensis sensu stricto* (S1); *P. restrepiensis* (PS3) showed high differentiation from other species (Corredor *et al.*, 2005; Matute *et al.*, 2006). PRP8 intein protein gene sequences from species of the *Paracoccidioides* spp. complex, including *P. restrepiensis* EPM83, were analyzed in a phylogenetic study. This gene can be used as a molecular marker since its polymorphism can separate species from the *P. brasiliensis* complex and *P. lutzii* (Theodoro *et al.*, 2008).

Some studies were directed to a phenotypic comparison of *P. restrepiensis* (PS3) isolates and other species belonging to the *Paracoccidioides* spp. (Table 2). Turissini *et al.* (2017) carried out a phenotypic study on yeast cells of cryptic species of *P. brasiliensis* complex (S1, PS2, PS3, and PS4) and *P. lutzii*. These authors observed that *P. restrepiensis* (PS3) has yeast cells larger than *P. brasiliensis sensu stricto* (S1) and *P. americana* (PS2) ones but no cell size differences with *P. venezuelensis* (PS4) and *P. lutzii* strains. A comparative study to evaluate PCM immunodiagnosis using species of the *P. brasiliensis* complex and *P. lutzii* showed higher GP43 production and best antigenic reactivity in an immunodiffusion assay with the EPM83 strain (*P. restrepiensis –* PS3) when tested with sera from patients living in a geographic area where *P. brasiliensis sensu stricto* (S1a e S1b) and *P. americana* (PS2) are prevalent (Machado *et al.*, 2013). Another comparative study of proteomes by disrupting yeast cells of *Paracoccidioides* species representative isolates showed that EPM83 (*P. restrepiensis* – PS3) preferentially expressed 38 proteins, including heat shock proteins (HSP) and a higher level of GP43 production (Pigosso *et al.*, 2013). An analysis of secretomes of two *Paracoccidioides* spp strains identified 95 extracellular proteins, 35 specific of *P. lutzii* and 36 specific of *P. restrepiensis* (PS3), including several ones related to fungal virulence factors and adhesion (de Oliveira *et al.*, 2018).

**Table 2:** Phenotypic characteristics of *P. restrepiensis* (PS3) isolates compared to other species of the *P. brasiliensis* complex and *P. lutzii*

*P. restrepiensis* isolate	P. brasiliensis complex or P. lutzii isolate	Evaluated trait	Observed differences	Study
C4, BAC, CNH	Pb18, T15LN1, T4B14 *- P. brasiliensis sensu stricto* (S1)	Yeast cell size	*P. restrepiensis* (PS3) shows larger yeast area than *P. brasiliensis sensu stricto* (S1) and *P. americana* (PS2) isolates but similar to *P. venezuelensis* (PS4)	(Turissini *et al*., 2017)
	Pb3 and T10B1 - *P. americana* (PS2)			
	Pb300 and Pb305 - *P. venezuelensis* (PS4)			
	Pb66, Pb01, EE - *P. lutzii*			
BAT	Pb18 - *P. brasiliensis sensu stricto* (S1)	Exoantigen production	Higher number of BAT exoantigens recognized by patient sera	(Panuto-Castelo *et al*., 2003)
	DGO and C – 9 - Unknown Genotype			
	B_339 – *P. brasiliensis sensu stricto* (S1)			
EPM83	Pb265 - *P. brasiliensis sensu stricto* (S1)	GP43 production and antigenicity	EPM83 showed higher GP43 production and antigenicity in the immunodiffusion test with PCM sera	(Machado *et al*., 2013)
	Pb01, Pb8334, Pb66 - *P. lutzii*			
EPM83	Pb339 – *P. brasiliensis sensu stricto* (S1)	Proteome	EPM83 presented higher expression of 38 proteins, including HSP and GP43	(Pigosso *et al*., 2013)
	Pb2 – *P.americana* (PS2)			
	Pb01 – *P. lutzii*			
EPM83	Pb01 - *P. lutzii*	Secretome	EPM83 production of 36 specific proteins; 21 proteins shared with Pb01	(de Oliveira *et al*., 2018)

B18 or Pb339 (ATCC32069) is a strain that has been reported in the literature to have divergent results regarding its classification as *P. restrepiensis* (PS3). It is originally from the state of São Paulo (Brazil) but from an unknown source. This strain was obtained from the National Communicable Disease Center (Atlanta, USA) and first studied by Restrepo-Moreno and Scheneidau JD in 1967. Matute *et al.* (2006) classified B18 as *P. brasiliensis sensu stricto* (S1b) by analyses of polymorphisms in nuclear genes, chitin synthase (CHS) 2, glucan synthase (FKS), *tub*1 (TUB), adenyl ribosylation factor (ARF), and *gp43* exon 2 loci. On the other hand, Salgado-Salazar *et al.* (2010) studied polymorphism of five mitochondrial genes used as markers in molecular characterization of *Paracoccidioides* species. The findings enabled reclassification of B18 strain as *P. restrepiensis* (PS3) from *Paracoccidioides* spp. complex (Salgado-Salazar *et al.*, 2010). Later, Roberto *et al.* (2016) evaluated B18 strain, termed in that study as B339 and /or EPM01 (ATCC200273) (Camargo *et al.*, 2003), and classified B18 strain as *P. restrepiensis* (PS3) by PCR-RFLP of *tub1* gene and sequencing of CHS2, FKS, TUB, ARF, and GP43 nuclear genes, the same ones studied by Matute *et al.* (2016). A study conducted by Turissini *et al.* (2017) classified B18 strain as an independent genotype due to its phylogenetic and micromorphological differences already observed in previous studies, suggesting a hybrid species belonging to the genus *Paracoccidioides*.

Identification of an unusual genotypic variant in southeastern Brazil contributes to understanding speciation and propagation involving PCM agents and may help in knowing *P. restrepiensis* characteristics (PS3 genotype) such as morphology, virulence, and serological reactivity.
